# The Central Inflammatory Network: A Hypothalamic fMRI Study of Experimental Endotoxemia in Humans

**DOI:** 10.1159/000519061

**Published:** 2021-10-05

**Authors:** Natalia Färber, Jorge Manuel, Marcus May, Nilufar Foadi, Florian Beissner

**Affiliations:** ^a^Somatosensory and Autonomic Therapy Research, Institute for Diagnostic and Interventional Neuroradiology, Hannover Medical School, Hanover, Germany; ^b^CRC Core Facility, Hannover Medical School, Hanover, Germany; ^c^Clinic for Anaesthesiology and Intensive Care Medicine, Hannover Medical School, Hanover, Germany

**Keywords:** Endotoxemia, Hypothalamus, Inflammation, Functional magnetic resonance imaging

## Abstract

**Introduction:**

Inflammation is a mechanism of the immune system that is part of the reaction to pathogens or injury. The central nervous system closely regulates inflammation via neuroendocrine or direct neuroimmune mechanisms, but our current knowledge of the underlying circuitry is limited. Therefore, we aimed to identify hypothalamic centres involved in sensing or modulating inflammation and to study their association with known large-scale brain networks.

**Methods:**

Using high-resolution functional magnetic resonance imaging (fMRI), we recorded brain activity in healthy male subjects undergoing experimental inflammation from intravenous endotoxin. Four fMRI runs covered key phases of the developing inflammation: pre-inflammatory baseline, onset of endotoxemia, onset of pro-inflammatory cytokinemia, and peak of pro-inflammatory cytokinemia. Using masked independent component analysis, we identified functionally homogeneous subregions of the hypothalamus, which were further tested for changes in functional connectivity during inflammation and for temporal correlation with tumour necrosis factor and adrenocorticotropic hormone serum levels. We then studied the connection of these inflammation-associated hypothalamic subregions with known large-scale brain networks.

**Results:**

Our results show that there are at least 6 hypothalamic subregions associated with inflammation in humans including the paraventricular nucleus, supraoptic nucleus, dorsomedial hypothalamus, bed nucleus of the stria terminalis, lateral hypothalamic area, and supramammillary nucleus. They are functionally embedded in at least 3 different large-scale brain networks, namely a medial frontoparietal network, an occipital-pericentral network, and a midcingulo-insular network.

**Conclusion:**

Measuring how the hypothalamus detects or modulates systemic inflammation is a first step to understand central nervous immunomodulation.

## Introduction

Inflammation is a mechanism of innate immunity that is part of the bodily reaction to pathogens, irritants, and injury. Since both, excessive and insufficient inflammation, can compromise the survival of the organism, it needs to be closely regulated by the body. Research advances of the past decades on the pathogenesis of inflammation have shown a complex bidirectional interaction between the central nervous system (CNS) and the innate immune system [1, 2, 3, 4]. The current understanding is that the immune system informs the CNS about inflammation or tissue injury in the body, which then sends efferent signals to control this inflammation via neuronal or neuroendocrine pathways [5, 6].

Immune signals can also act directly on the CNS, thus influencing neuronal activity. For example, cytokines can stimulate the CNS after crossing the blood-brain barrier via active transport [7]. Furthermore, neurons of the circumventricular organs are able to sense the composition of the blood and detect bacterial endotoxin or the cytokines it induces [8]. Some pro-inflammatory cytokines can also act as signalling molecules through cytokine-specific receptors located in brain tissue [9, 10]. Peripheral inflammation causes a mirror inflammatory response in the CNS, characterized by additional synthesis of cytokines within the brain [11, 12]. Moreover, viscero-sensory fibres of the vagus nerve are able to detect bacterial products, cytokines, and other inflammatory mediators [13], whereas silent mechano-insensitive nociceptors of Aδ- and C-fibres can sense local inflammation through pain signals [14, 15].

The fever response, one example of how the innate immune system influences the CNS, is carried out by integrated neuronal and physiological circuitry. Macrophages and dendritic cells release prostaglandin E2 (PGE2) and pyrogenic cytokines (interleukin-1β [IL-1β], interleukin-6 [IL-6], and tumour necrosis factor [TNF]) that act systemically to induce fever [16]. During endotoxemia specifically, PGE2 is produced by brain vascular endothelial cells of the hypothalamic median preoptic area and considered to be a major pyrogenic mediator of fever [17, 18, 19]. PGE2 triggers the sympathetic branch of the autonomic nervous system, which raises body temperature by increasing thermogenesis in brown adipose tissue and inducing vasoconstriction to prevent passive heat loss [20, 21].

In contrast, the most well-established pathway of immunomodulation by the CNS is the hypothalamic-pituitary-adrenal (HPA) axis. Circulating cytokines, in particular TNF, activate neurons in the hypothalamus that trigger the secretion of corticotropin-releasing hormone (CRH) into the primary capillary plexus of the hypothalamo-hypophyseal portal system. CRH stimulates the anterior lobe of the pituitary gland to secrete adrenocorticotropic hormone (ACTH) into the bloodstream [22, 23]. Finally, ACTH leads to a release of cortisol and other glucocorticoid hormones from the adrenal medulla that control innate immune cells by suppressing pro-inflammatory cytokine synthesis [24].

In addition to the HPA axis, the last 2 decades of research have identified several new pathways, by which the CNS controls inflammation. All of them are based on direct neural modulation of immune cells or immune organs and are mediated by the ANS [3, 4, 25]. Although their building blocks have been the subject of intensive research, our understanding of these pathways remains fragmentary. In particular, central nervous mechanisms underlying ANS-mediated immunomodulation are almost completely unknown. For example, Chavan and Tracey [5] call interactions of afferent and efferent signals in the CNS a black box. Jänig [14] states that the central circuits are largely unknown for most pathways involved in regulation of inflammation. Since the efferent pathways of immunomodulation by the ANS are the same that regulate other body functions, such as cardiovascular or digestive functions, it is very likely that the nervous structures controlling immunomodulation are similar to those regulating other body functions. Thus, recent studies on systemic inflammation have shown that the relatively well-established centres in the hypothalamus that control ANS activity are overlapping with those modulating immunity [26, 27, 28].

Therefore, we investigated inflammation-associated regions of the central nervous system in humans by combining functional magnetic resonance imaging (fMRI) with the human endotoxemia model (HEM) [29, 30]. We focused on functional centres in the hypothalamus, while continuously measuring serum levels of ACTH and TNF. We further sought to differentiate regions that detect cytokinemia from those controlling HPA axis-related activity, by testing the blood oxygenation level dependency (BOLD) signal of the hypothalamic nuclei for temporal correlation with the serum level time courses of TNF and ACTH. Finally, we studied the connection of these inflammation-associated hypothalamic subregions with the rest of the brain to assess how they fit in the context of large-scale brain networks.

## Materials and Methods

The study took place at Hannover Medical School and was conducted in accordance with the Declaration of Helsinki. It was approved by the Local Ethics Committee (IORG0002700, approval No. 7427). Seven healthy, normotensive men (26.0 ± 8.5 years, 24.6 ± 2.6 kg/m^2^) took part in the study. All participants gave written informed consent including consent to publish their data anonymously. We informed the participants that they would receive an endotoxin or saline injection at some point during the fMRI experiment. However, all subjects received the endotoxin with the start of the second fMRI acquisition.

General exclusion criteria included a body mass index of <18 and >30 kg/m^2^, any concurrent medical condition, history of allergies, current use of prescription and non-prescription medications, smoking, and regular high alcohol use. To exclude any inflammatory diseases that may aggravate through the HEM, each subject was interviewed and examined by a physician before being admitted to the study. A brief physical examination was also performed on the day of the study. Furthermore, blood parameters were checked the day before, right before the experimental procedure, and the day after completion of the study. All subjects received a remuneration of 30 EUR/h for their participation.

### General Experimental Design

**Fig. 1:**
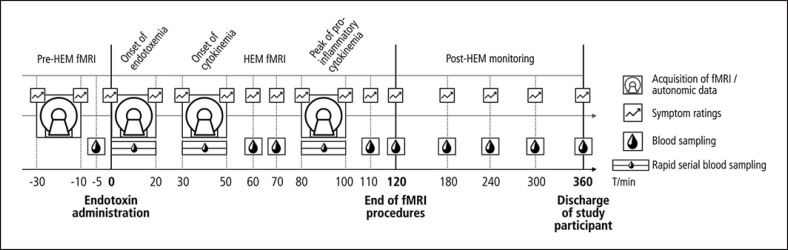
Experimental design. The experiment consisted of 3 periods: (1) pre-HEM-fMRI with baseline measurements of fMRI, symptom ratings and blood parameters, (2) HEM-fMRI with 3 combined measurements of fMRI and rapid serial blood sampling covering the onsets of endotoxemia and the onset and peak of pro-inflammatory cytokinemia, and (3) post-HEM monitoring, during which subjects were transferred to a clinical unit for further monitoring and discharged 6 h after injection, when all illness symptoms and pro-inflammatory cytokines had returned to their normal values. HEM, human endotoxemia model; fMRI, functional magnetic resonance imaging.

Experimental procedures comprised an fMRI experiment of 2 and a half hours followed by a period of 4–6 h, during which the participants were monitored. We focused our fMRI measurements on the first 2 h after endotoxin administration (HEM-fMRI) to cover the most relevant physiological processes, while limiting the participants' burden associated with the MRI measurements. Separate resting-state fMRI measurements, each of 20 min length, were conducted at 4 relevant time points: (1) between 30 and 10 min before endotoxin administration (“baseline”), (2) from the moment of injection to 20 min after, that is, at the onset of endotoxemia, (3) around the onset of pro-inflammatory cytokinemia, that is, from 30 to 50 min after injection, and (4) around the peak of pro-inflammatory cytokinemia and illness symptom severity according to previous studies [29, 30, 31, 32], that is, from 80 to 100 min after injection (shown in Fig. 1).

### Human Endotoxemia Model

All subjects received an intravenous bolus injection of endotoxin over 1 min through an intravenous catheter in an antecubital forearm vein. GMP-grade lipopolysaccharide from *Escherichia coli* O:113:H10:K-strain (Lot 94332B1) provided by National Institute of Health Clinical Center, Bethesda, MD, USA, was prepared for human use by reconstitution with sterile water for injection, shaking for 15 min on a vortex shaker and final dilution. We used a dose of 1 ng/kg (0.02 mL/kg) body weight, since the dose was sufficient to provide an innate immune response, while avoiding unnecessary overreaction to endotoxin [33]. In particular, we wanted to avoid that subjects started shivering during the MRI, as excessive head motions would make hypothalamic fMRI impossible. Furthermore, we could keep subjects blinded because they were told that they would receive either endotoxin or a placebo. All endotoxin solutions were administered immediately after their preparation. Subjects were injected between 9 and 10 o'clock in the morning and discharged 6–8 h later, when their symptoms had receded, all altered physiological parameters had demonstrated consistent reduction towards baseline values, and the physical exam was normal.

### Blood Sampling and Analysis

The analysis of blood parameters is the most direct way to assess immune system activity. As our main goal was to detect neural activity related to the inflammatory process, we required a fast marker with a short half-life. Thus, TNF and IL-1β were the only 2 candidate markers to monitor the rapidly changing inflammatory process as they are released early during inflammation [34, 35, 36, 37]. We chose TNF, a pro-inflammatory cytokine chiefly produced by activated macrophages in response to endotoxin, as it can be measured more reliably than IL-1β [37, 38]. In contrast, other cytokines, like IL-6, are released with considerable delay and are therefore unsuitable for this purpose [32, 36]. To assess HPA axis activity, we chose ACTH because it is more closely linked to the neural part of the HPA axis than cortisol itself. Both parameters were analysed from venous blood serum using an enzyme-linked immunosorbent assay for TNF (EIA-4641; DRG Instruments GmbH, Marburg, Germany) and electrochemical luminescent immunoassay for ACTH (COBAS e801 Immunoassay System; Roche Diagnostics GmbH, Mannheim, Germany) following standard protocols.

Since the time course of HEM-related immune activation is well known from multiple studies [29, 30, 31, 32], we limited the assessment of blood parameters to the most relevant time points. Thus, samples were collected every 30 s during the 3 fMRI runs of the HEM-fMRI period, every 10 min between the runs, and every 60 min in the post-HEM monitoring period (shown in Fig. 1). Blood samples were taken manually from the i.v. line that was in place throughout the entire experiment. The procedure was timed with a normal clock visible by the experimenter inside the MRI chamber. Samples from the fMRI runs were later combined by pouring each 2 successive blood samples together resulting in an effective sampling rate of 1 per minute. It is important to note that the total blood volume drawn in the study was <200 mL (126 vials of 1 mL each), which is well below a standard blood donation of 450 mL. To avoid blood sampling problems due to vasoconstriction, we kept the subjects' hands warm during the study using 0.5 L PET bottles with warm water (well below the pain threshold).

### Symptom Ratings and Autonomic Recordings

Participants were asked to rate their illness symptoms (headache, shivering, muscle ache, nausea, and fatigue) and anxiety at different times during the HEM (shown in Fig. 1). We used numeric rating scales ranging from 0 (non-existent) to 10 (worst severity imaginable). A symptom score was calculated as the sum of all 6 illness symptoms, meaning that it could take on values between 0 and 6. Body temperature was measured orally using a standard electronic thermometer before every symptom rating.

To monitor the subjects' vital signs and autonomic nervous system activity, we measured pulse (photoplethysmography), respiratory frequency and amplitude (respiratory belt), and blood pressure (digital artery) during the entire HEM-fMRI period using an MR-compatible BIOPAC MP150 data acquisition system (BIOPAC Systems Inc., Goleta, CA, USA). These measurements were mainly taken for safety reasons. Only blood pressure values are presented in the results. Blood pressure was measured using a validated pulse decomposition analysis, which reconstructs blood pressure from the amplitude and time lags of reflected pulse waves [39].

### MRI Data Acquisition and Preprocessing

All measurements were taken on a Siemens 3T MAGNETOM Skyra (Siemens Healthineers, Erlangen, Germany), a whole-body MR scanner with a 64-channel head/neck coil. Participants were instructed to relax with their eyes closed. The measurement comprised a functional whole-brain gradient-echo echo-planar imaging (EPI) sequence (TR = 1,180 ms; TE = 32 ms; simultaneous multi-slice factor = 6; partial Fourier = 7/8; 2 mm isotropic resolution). We acquired 450 volumes at baseline and 900 volumes at each of the other 3 measurements. We further acquired reference scans for motion correction and template formation that were equivalent to the functional sequence but without multiband acceleration (TR = 6,770 ms), as well as reference scans for unwarping purposes consisting of 2 spin-echo images that were distortion-matched to the functional scans but without multiband acceleration. Of the latter, one was acquired with the same direction and the other one with inverted phase-encoding direction. Finally, we acquired structural scans using a T1-weighted magnetization-prepared rapid acquisition gradient-echo image sequence (TR = 2,400 ms; TE = 2.13 ms; TI = 1,000 ms; in-plane acceleration factor = 2; and isotropic resolution: 1 mm).

fMRI data preprocessing used tools from FSL v5.0.9 (FMRIB Software Library; University of Oxford, Oxford, UK, http://fsl.fmrib.ox.ac.uk). The individual steps were motion correction (linear realignment of functional images to the gradient-echo EPI reference scan, MCFLIRT [40]) and unwarping (estimation of magnetic field inhomogeneities from the spin-echo reference images with inverse phase-encoding directions, TOPUP [41]) applied in a single transformation, ICA-denoising (manual classification of components applying criteria shown in [42], then regression of noisy time courses only), brain extraction [43], grand mean scaling, and high pass filtering (0.005 Hz).

We generated 2 study templates using Advanced Normalization Tools (https://www.nitrc.org/projects/ants [44]), one from the unwarped gradient-echo EPI reference images, and the other from the T1 images. For group analyses, we transformed the functional images to the T1 template in a single transformation. This procedure included a symmetric diffeomorphism to the EPI-template followed by a 6-degrees-of-freedom transformation to the T1 template. Finally, we transformed the results to the 6th-generation non-linear Montreal Neurological Institute standard space (MNI152 [45]) using a non-linear transformation (Advanced Normalization Tools). All transformations used linear interpolation during resampling. The data for whole-brain analyses were smoothed using a Gaussian kernel of 5 mm full width at half maximum. In contrast, all hypothalamic analyses used unsmoothed data.

### Statistical Analysis

To assess systemic inflammation, we compared the serum levels of ACTH and TNF, as well as the number of leukocytes at baseline (minute 0 for ACTH and TNF, −5 min for leukocytes) to their values at 120 min using a 2-tailed paired *t* test. The same test was used to assess changes in body temperature and symptom severity. A *p* value of 0.05 was considered significant.

For the fMRI data, we first segmented the hypothalamus into 35 functionally distinct regions using masked independent component analysis ([46, 47]) on the concatenated functional data of all subjects with a hypothalamic mask based on the anatomical atlas of Mai et al. [48]. The ICA dimensionality was derived by visual examination of the functional segmentations between 2 and 50 components. We chose the first dimensionality that showed the correct anatomical subdivisions known from the literature [49]. Independent components were tested for specificity by running an unmasked dual regression [50] using their spatial maps as input and the whole brain as target region and then calculating the weighted quotient of activation in grey matter versus white matter and cerebrospinal fluid using probabilistic masks obtained with FAST [51]. Components were considered unspecific if this quotient was <1 standard deviation from the mean [46, 52].

The remaining components were considered to reflect inflammation-associated hypothalamic subregions if they fulfilled the following 2 criteria: (1) components showing significant temporal correlation with either TNF or ACTH time courses (Bonferroni corrected). To calculate the temporal correlation of BOLD signals and ACTH and TNF serum levels, we downsampled both time courses to 10 data points by averaging BOLD signals over 300-volume blocks and serum levels over 12 consecutive blood samples. The averaged final 300 volumes of the baseline run and the serum levels from the baseline blood sample served as the first data point. (2) Components showing a significant change of within-hypothalamus functional connectivity, when comparing the individual HEM-fMRI runs. Changes in functional connectivity were calculated by means of dual regression analysis within the hypothalamic mask. Significance was assessed using a permutation-based, non-parametric, paired *t* test thresholded at *p* < 0.05 using family-wise error (FWE) correction with threshold-free cluster enhancement (TFCE). Due to the small sample size of the study, these data were only used for identification but are not shown.

In a final analysis, we derived whole-brain functional connectivity profiles of all inflammation-associated hypothalamic subregions by using an unmasked dual regression from the masked region to the rest of the brain. Here, significance was assessed using a permutation-based, non-parametric, one-sample *t* test, thresholded at *p* < 0.01 (FWE correction with threshold-free cluster enhancement). To identify similarities between the whole-brain connectivity profiles and to aid their characterization, we calculated the spatial correlation matrix of the unthresholded maps using FSLCC. Using R [53] with gplots [54], we then plotted the results in a heat map with a dendrogram showing complete-linkage clustering based on the Euclidean distance.

## Results

### Time Course of Inflammatory Activity

**Fig. 2:**
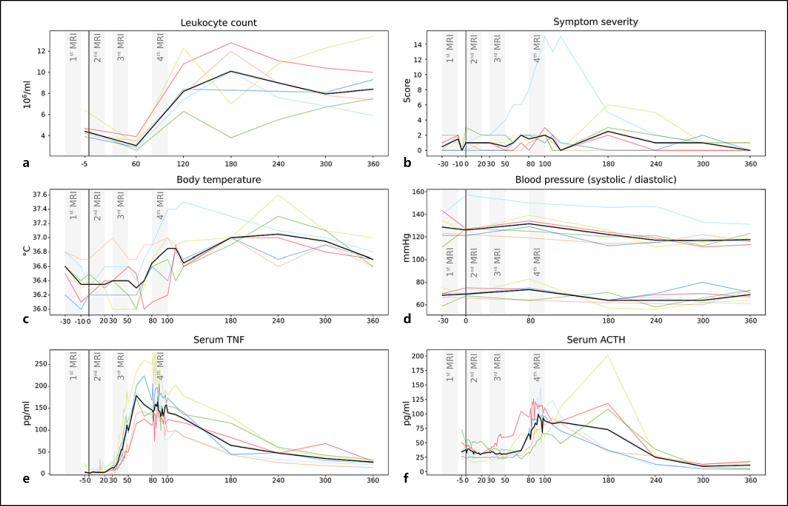
Time courses of inflammatory activity. Each plot shows individual curves for the 6 subjects (coloured lines) and the median (black line). At 0 min (vertical black line), all subjects received a bolus injection of endotoxin triggering a systemic inflammatory response. As expected, the 3 fMRI runs (grey bars) after the pre-injection baseline (1st), coincided with the onset of endotoxemia but without showing TNF nor ACTH activity (2nd), the onset of pro-inflammatory cytokinemia with TNF but not ACTH increase (3rd), and peak pro-inflammatory cytokinemia with TNF and ACTH at their maximum levels (4th). Note that body temperature increase and symptom severity were not pronounced except for one subject. **a** Leukocyte count (10^6^/mL); **b** symptom severity (score); **c** body temperature (°C); **d** blood pressure (systolic/diastolic, mm Hg); **e** serum TNF (pg/mL); and **f** serum ACTH (pg/mL). TNF, tumour necrosis factor; ACTH, adrenocorticotropic hormone; fMRI, functional magnetic resonance imaging.

All subjects showed signs of systemic inflammation following experimental endotoxemia (shown in Fig. 2). In particular, we observed a significant increase in serum TNF (110.9 ± 37.7 pg/mL, *p* = 0.0029), ACTH (42.8 ± 38.1 pg/mL, *p* = 0.0388), and in the number of leukocytes (4.2 ± 1.7∙10^6^/mL, *p* = 0.0017), between baseline (pre-HEM) and 120 min (end of HEM-fMRI). However, neither body temperature nor symptom severity showed significant differences. One subject was excluded from further analysis due to signs of an ongoing inflammation before endotoxin injection (leukocyte count: 12.4∙10^6^/mL). One subject developed strong flu-like symptoms and a peak body temperature of 37.5°C.

TNF, ACTH, and leukocyte count all showed time courses indicative of a limited systemic inflammation (shown in Fig. 2). Leukocyte concentration showed a characteristic drop at 60 min followed by a strong increase in all subjects at 120 min (shown in Fig. 2a). TNF concentration at baseline ranged near zero and started increasing after 20–30 min reaching a peak at 60–70 min, after which it started returning to normal values (shown in Fig. 2e). The initial ACTH increase was approximately 20–30 min delayed compared to that of TNF. Peak serum concentrations were reached between 100 and 180 min, and values had returned to baseline values at 240 min (shown in Fig. 2f).

### Functional Hypothalamic Centres

**Fig. 3:**
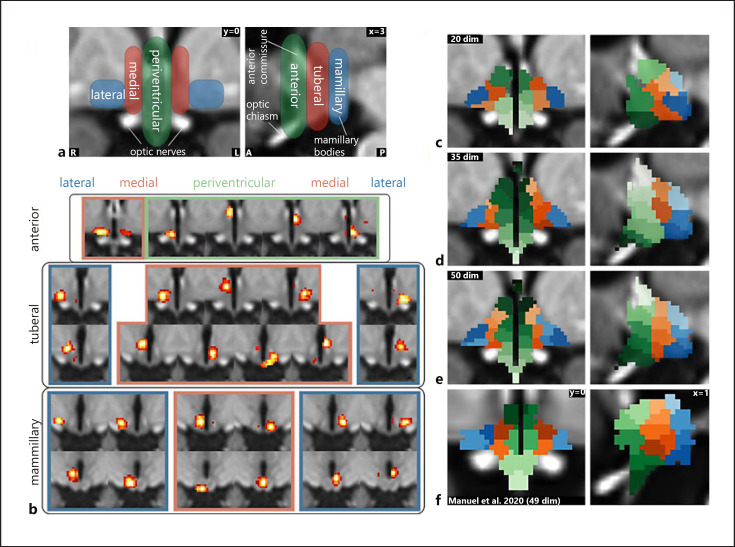
Functional segmentation of the human hypothalamus. **a** Anatomical subdivisions of the human hypothalamus based on post-mortem anatomical studies (Dudás [49]). Left: Hypothalamic zones in the mediolateral direction. Right: Hypothalamic regions in anterior-posterior direction. **b** Complete set of specific spatially independent components as used for connectivity-based analyses in this study. Not shown are 7 components that were identified as noise. **c–e** Segmentation into functionally independent subregions comparing 3 different dimensionalities based on the results of a masked ICA of the functional MRI data: Twenty-dimensional segmentation showing 3 zones and 3 regions but with a significant portion of the hypothalamus not included in the segmentation (**c**); thirty-five-dimensional segmentation showing all 3 zones and regions with further subdivisions in superior-inferior direction (**d**); fifty-dimensional segmentation showing overfitting (at least 4 zones on each side) (**e**); and **f** segmentation from an independent dataset of 22 healthy subjects using a smaller mask and 49 dimensions; modified from Manuel et al. [52] under CC BY 4.0.

A 35-dimensional segmentation of the hypothalamus yielded the characteristic 3 mediolateral zones and 3 anterior-posterior regions (shown in Fig. 3a, d) as expected from post-mortem anatomical studies [49] and a previous fMRI study using similar methodology [55] (shown in Fig. 3f). Seven components were classified as noise and excluded from further analysis. The remaining components are shown in Figure 3b.

**Fig. 4:**
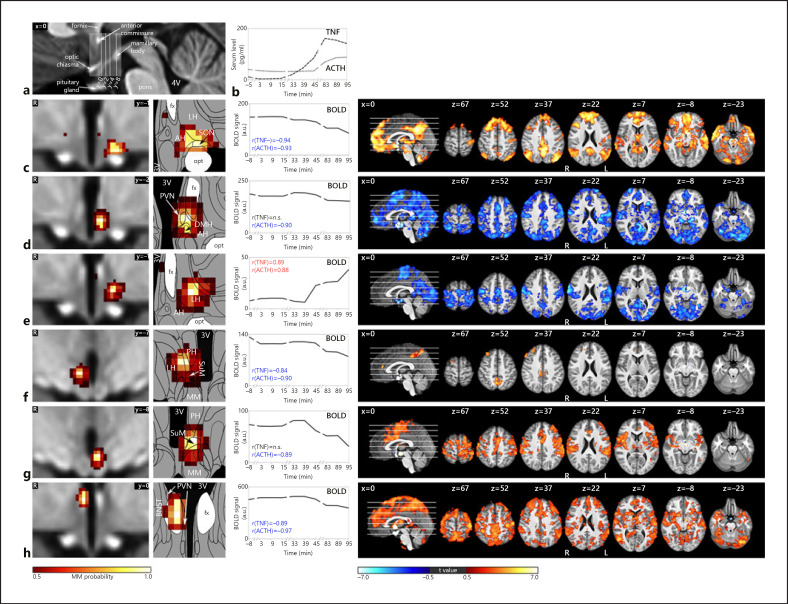
The central inflammatory network. **a** T1-weighted study template showing slice localization in the hypothalamus. **b** Group mean time-courses of TNF and ACTH serum concentrations during HEM-fMRI rapid serial blood sampling. **c–h** Left: Individual hypothalamic centres derived from the mICA presented as mixture model thresholded probability maps. Centre: Average BOLD signal of the respective centres. Right: Cortical connectivity of the immunomodulatory hypothalamic regions. Functional connectivity maps of the respective region (*p* < 0.01, FWE corr.). Warm/cold colours indicate a correlation/anticorrelation in cortical connectivity during HEM. Every identified hypothalamic centre shows either correlation or anticorrelation with time courses of TNF and ACTH serum concentration and positive or negative functional cortical connectivity. All coordinates are in MNI152 standard space. 2016 Academic Press, Elsevier. All rights reserved. Atlas slices modified from Mai et al. [48]. 3V, third ventricle; AH, anterior hypothalamic area; BNST, bed nucleus of the stria terminalis, DMH, dorsomedial hypothalamic nucleus; fx, fornix; LH, lateral hypothalamus; MM, mammillary bodies; opt, optic tract; PVN, paraventricular nucleus; PH, posterior hypothalamic area; SuM, supramammillary nucleus; SON, supraoptic nucleus; fMRI, functional magnetic resonance imaging; mICA, masked independent component analysis; HEM, human endotoxemia model; BOLD, blood oxygenation level dependency; TNF, tumour necrosis factor; ACTH, adrenocorticotropic hormone; MNI, Montreal Neurological Institute.

Of these 28 components, 6 satisfied both criteria for inflammatory relevance (shown in Fig. 4c-h; Table 1). Four showed significant correlations with the TNF and all 6 with the ACTH time course. Correlation values were very high (0.84 < *r* < 0.97) and negative for all subregions except for the lateral hypothalamic (LH) area, which showed a positive correlation (shown in Fig. 4e).

The first hypothalamic subregion (shown in Fig. 4c) showed BOLD signal correlation with both TNF and ACTH. It was located in the anterior region of the hypothalamus and comprised the supraoptic nucleus (SON), LH area, and anterior hypothalamic (AH) area. The second subregion (shown in Fig. 4d) consisted of the left tuberal paraventricular nucleus (PVN) and dorsomedial hypothalamic nucleus (DMH), showing negative BOLD signal correlation with ACTH but not TNF. The third hypothalamic subregion (shown in Fig. 4e) was located in the tuberal region comprising the left LH and AH and showing positive BOLD signal correlation with both TNF and ACTH. Subregion 4 (shown in Fig. 4f) comprised the right supramammillary nucleus (SuM), posterior hypothalamus and LH. It showed negative BOLD signal correlation with both TNF and ACTH. The fifth subregion (shown in Fig. 4g) consisted of the left SuM and parts of the mammillary bodies and posterior hypothalamus. It showed negative BOLD signal correlation with ACTH. Finally, the sixth hypothalamic subregion (shown in Fig. 4h) was centred in the right fornix, but extended into the bed nucleus of the stria terminalis (BNST) and the PVN. Its BOLD signal was negatively correlated with both TNF and ACTH.

**Fig. 5:**
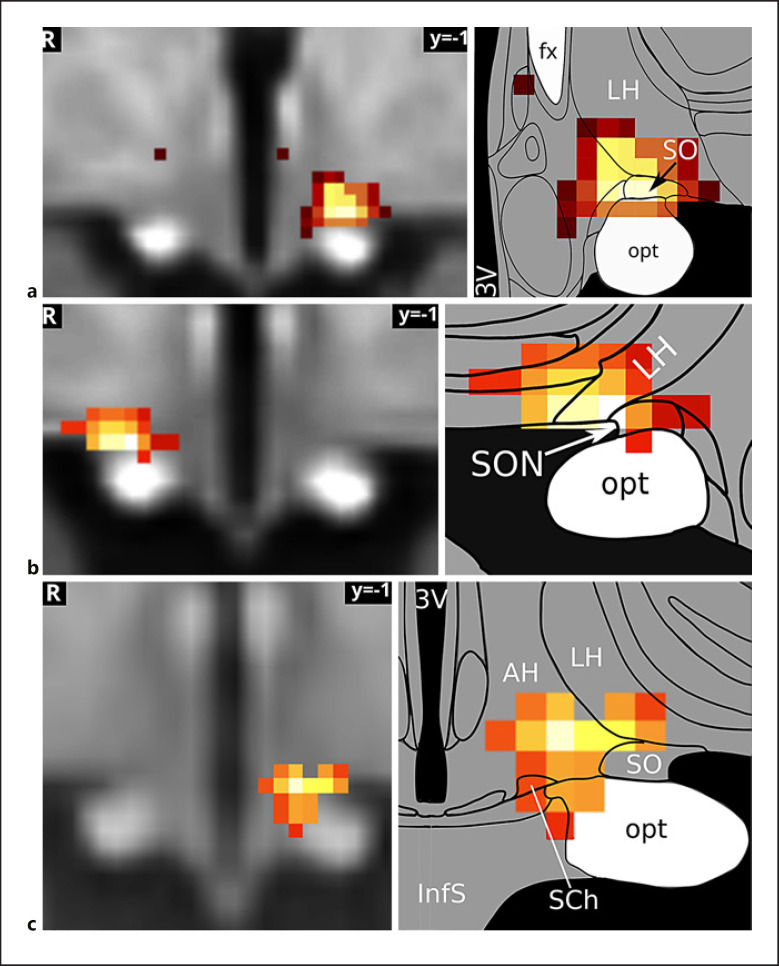
The supraoptic nucleus/LH area in autonomic and immune regulation. The hypothalamic subregion associated with the medial frontoparietal network and negatively correlated with both TNF and ACTH (**a**) has previously been shown to mediate blood pressure changes related to baroreflex (Manuel et al. [52]) (**b**) and chemoreflex stimulation (Gerlach et al. [55]) (**c**). It may therefore play a central role in immunomodulation by the autonomic nervous system. The association of the medial frontoparietal network with parasympathetic autonomic regulation (Beissner et al. [57]) gives further support to this hypothesis. Image sources are modified after Manuel et al. [52] under CC BY 4.0 (**b**) and modified after Gerlach et al. [55] (**c**). Atlas slices modified from Mai et al. [48]. 3V, third ventricle; fx, fornix; LH, lateral hypothalamus; opt, optic tract; SON, supraoptic nucleus; TNF, tumour necrosis factor; ACTH, adrenocorticotropic hormone; LH, lateral hypothalamic.

A comparison of the first inflammation-associated hypothalamic subregion with results from our previous studies that had used similar methodologies revealed very similar regions comprising SON, LH, and AH (shown in Fig. 5). This is interesting, as these studies did not study the effects of inflammation, but used orthostatic stress [55], and hypoxia [56] to study blood pressure regulation by the autonomic nervous system.

### Characterization of Large-Scale Brain Networks

**Fig. 6:**
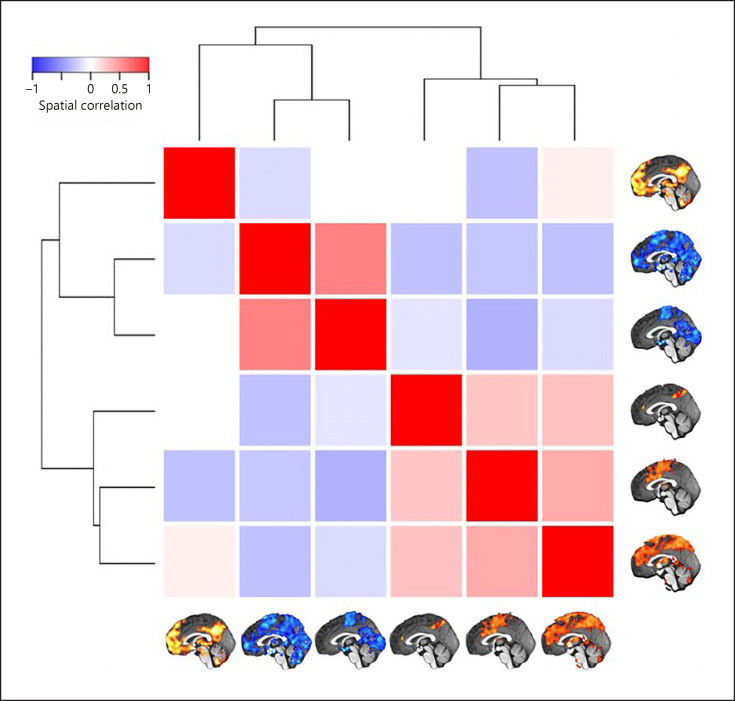
Similarity of whole-brain connectivity networks. The matrix shows the spatial correlation of the 6 whole-brain networks associated with the hypothalamic subregions involved in inflammation (Fig. 4c–h). Unthresholded maps were used for the procedure. The dendrogram quantifies the networks' dissimilarity after complete-linkage clustering based on Euclidean distance. As the result shows, there are at least 3 clusters of similar networks, the first containing only the medial frontoparietal network, the second containing 2 occipital-pericentral networks, and the third containing the midcingulo-insular and 2 similar networks.

Whole-brain connectivity networks of the inflammation-associated hypothalamic subregions were not mutually orthogonal but roughly formed 3 clusters of networks (shown in Fig. 6). To describe them, we follow the nomenclature proposed by Uddin et al. [56]. Anatomical regions of each network are shown in Table 2.

The network that positively correlated with the first hypothalamic subregion was the medial frontoparietal network (M-FPN), also known as default mode network. As the dendrogram shows, this network was dissimilar from all other networks. Whole-brain connectivity for the second and third hypothalamic subregions was both negative, and the 2 networks formed the second cluster showing the highest similarity of all networks (*r* = 0.50). In terms of large-scale brain networks, both were mixtures of pericentral (also known as sensorimotor) and occipital (also known as visual) networks. The final 3 networks formed the third cluster with correlation coefficients ranging from 0.24 to 0.33. Their connectivity with the hypothalamic subregions was positive. While the network of the fourth hypothalamic subregion was sparse including only precuneus cortex, anterior cingulate gyrus, and right frontal pole, the networks of the fifth and sixth subregions were widespread sharing pre- and postcentral, insular, anterior cingulate, central opercular, and supramarginal regions. We identified the fifth network as the midcingulo-insular network (M-CIN) (also known as salience or ventral attention network).

## Discussion

We investigated central nervous regions associated with inflammation in humans by combining functional MRI with the HEM. Our results show that there are at least 6 inflammation-associated hypothalamic subregions including the PVN, SON, bed nucleus of the stria terminalis, dorsomedial hypothalamus, LH area, posterior hypothalamic area, and supramammillary nucleus. These regions are functionally embedded in at least 3 different large-scale brain networks, namely the M-FPN, M-CIN, and an occipital-pericentral network.

Regarding the individual regions, the PVN (shown in Fig. 4d, h) is known to play a pivotal role in immune responses, in particular, regarding the activation and modulation of the HPA axis after endotoxin injection [58, 59, 60, 61]. Neurons of the parvocellular PVN synthesize CRH under both physiological and pathological conditions and release it into portal vessels of the median eminence that reach the anterior pituitary, where CRH stimulates the synthesis and release of ACTH into the bloodstream [22, 23, 62]. When ACTH reaches the adrenal cortex, it stimulates the synthesis and secretion of glucocorticoids [63], which in turn regulate the synthesis of CRH and the ACTH precursor proopiomelanocortin in a negative feedback loop mediated by glucocorticoids receptors in the PVN, anterior pituitary, hippocampus, amygdala, and prefrontal cortex [64, 65]. TNF, as one of the potent pro-inflammatory cytokines, can activate the HPA axis through indirect stimulation of PVN neurons [27]. Recently, it was shown that TNF is also a signalling molecule that directly acts upon the PVN and groups of neurons in the limbic forebrain through TNF receptor of type 1, whose activation increases excitatory synaptic strength and inhibits GABAergic neurons [10].

The SON (shown in Fig. 4c) is an important component of the neural pathway controlling autonomic, neuroendocrine, and behavioural responses to diverse homeostatic challenges, including emotional stressors, such as restraint stress, and pathophysiological processes, such as inflammation [66]. Hare et al. [67] showed that an increase in plasma ACTH after endotoxin administration resulted in an activation of neurons in the hypothalamic SON, PVN, and BNST as indicated by the enhanced expression c-Fos. Qadri et al. [66] showed that after endotoxin injection, the HPA axis is activated by means of pro-inflammatory mediators bradykinin and des-Arg9-bradykinin acting via bradykinin-1 receptors located on SON and bradykinin-2-receptors located on PVN and SON. Such specific localization indicates that PVN and SON play a crucial role in the maintenance of body homeostasis during endotoxemia.

Furthermore, histologically the SON is magnocellular and produces the 2 peptide hormones vasopressin and oxytocin [68]. It is 1 of the 2 major nuclei of the hypothalamo-neurohypophyseal system that is tightly linked to the HPA axis [69, 70]. For example, there is abounding evidence demonstrating the involvement of magnocellular vasopressin in the direct control of ACTH secretion [70]. Moreover, peripheral immunoneutralization of oxytocin attenuated the increase in plasma ACTH, suggesting an involvement of oxytocin in the regulation of ACTH secretion [71]. Neuro-anatomical studies have also demonstrated that the SON exhibits reciprocal connections with several limbic forebrain regions involved in autonomic and behavioural responses, such as the lateral septum, the ventral and dorsal hippocampus, BNST, the amygdaloid complex, and cortical regions [72, 73]. Two of our previous studies have confirmed the role of the SON in cardiovascular regulation in humans in response to orthostatic stress [52] (shown in Fig. 5b) and hypoxia [55] (shown in Fig. 5c). Further support for this double role of the SON comes from its association with the M-FPN discussed below.

The DMH (shown in Fig. 4d) is involved in the control of both endotoxin-induced hypothermia [74] and fever [75]. The DMH is also involved in thermoregulatory cutaneous vasoconstriction, shivering, and endocrine adjustments [75]. While Ebner et al. [76] stated that the DMH is involved in HPA axis activation only in response to an emotional stressor but not to immune challenge, our results show that DMH is also associated with inflammatory response in humans. This is supported by Hunt et al. [77], who demonstrated that neurons in the DMH play a key role in the activation of the HPA axis evoked from the activity of the medial preoptic area, the main region responsible for the fever response during inflammation [16]. Furthermore, the DMH is a target for the rapid actions of corticosteroids [78] and plays a role in negative feedback control on stress-induced HPA axis activation [79] via glutamatergic and GABAergic projections to the parvocellular neurons in the PVN [77, 80].

The SuM (shown in Fig. 4f, g) may play an important role in modulating inflammation. Furube et al. [81] showed that the single endotoxin stimulation of mice at a high dose of 1 mg/kg induced microglial proliferation in numerous brain regions, including SuM, PVN, SON, and LH, taking into account that microglia are the main producers of inflammatory mediators in the brain and play a principal role in innate immunity [82]. In addition, the majority of CRH-immunoreactive cells have been found to colocalize in the SuM and have their projections to the hippocampus [83]. The hippocampus in turn plays a pivotal role in the glucocorticoid-mediated negative feedback of the HPA axis [84].

The BNST (shown in Fig. 4h), as a key relay connecting limbic forebrain structures to the hypothalamus, has projections to the parvocellular PVN that participate in activation of the HPA axis [85, 86, 87]. However, anatomical studies indicate that the vast majority of BNST input to the parvocellular PVN is GABAergic [88], suggesting that the BNST provides largely inhibitory modulatory input to the HPA axis [89]. Moreover, Fukuwada et al. [90] recently presented evidence for a role of the BNST in regulating endotoxin-induced despair-like behaviour in mice via Gαq protein signalling. Finally, the BNST is involved in control of inflammation-associated loss of appetite [91].

The LH (shown in Fig. 4e) plays a significant role in the regulation of inflammatory pain, providing analgesic and anti-nociceptive effects [92, 93]. These effects are largely mediated by inhibiting descending pathways from the hypothalamus to the spinal cord through the release of oxytocin synthesized by the PVN and SON, which suppresses nociception and promotes analgesia [94]. Consistently, LH has been shown to receive dense nociceptive inputs from the spinothalamic tract, which convey information related to pain [95]. Previous studies indicate that the LH, in conjunction with the HPA axis, coordinates inflammation in the CNS [28]. Orexin, leptin, and GABA neurons of the LH play a pivotal role in balancing the HPA axis, either directly or indirectly by acting on intermediate structures [96], whereas activation of CRH receptors leads to the excitation of orexin neurons in the LH [97].

As the previous sections show, our results regarding the set of nuclei associated with systemic inflammation are largely in line with animal studies. However, there is a seeming discrepancy between the known activation of most of these nuclei during systemic inflammation and the observation that their BOLD signal changes showed negative correlations with TNF and ACTH. We believe that this discrepancy is due to limitations of hypothalamic fMRI rather than inter-species differences. The BOLD signal measured in fMRI is formed by the time courses of cerebral blood flow and the cerebral metabolic rate of O_2_ (CMRO_2_), both of which correlate strongly with changes in the electrical brain activity [98]. Importantly, BOLD signals are influenced more by subthreshold synaptic input than by axonal output, and negative BOLD signals under most conditions correlate to the excitation of inhibitory interneurons [99]. Furthermore, while CMRO_2_ increases are controlled by ATP turnover, which depends on the energy used to fuel the Na⁺/K⁺-ATPase to reestablish ionic gradients, cerebral blood flow increases are mainly controlled by Ca^2+^-dependent mechanisms in neurons and astrocytes. This explains why upon increased neuronal activity, we may see increases, decreases, or no BOLD signal changes in the fMRI [98]. Thus, it is not possible based on a negative or positive BOLD signal alone to decide whether the underlying activity goes on in principal or inhibitory neurons [98].

Regarding the involvement of extra-hypothalamic regions, our results show that at least 3 large-scale brain networks, namely the M-FPN, M-CIN, and occipital-pericentral network are associated with inflammation in humans. This is in line with previous research by other groups. For example, the M-FPN, M-CIN, occipital, and dorsal frontoparietal networks all show increased connectivity to the left inferior parietal lobule at higher levels of peripheral inflammation [100]. Furthermore, a recent meta-analysis has shown the involvement of the M-FPN and M-CIN in inflammation [101]. Labrenz et al. [102] showed that systemic inflammation affects multiple regions within the human brain at rest. They found that after endotoxin injection functional connectivity was increased between the left thalamus and cerebellum, while the functional connectivity within multiple and widespread networks originating from the amygdala, insula, and cingulate cortex was reduced. Lekander et al. [103] found an increase of connectivity between the left anterior insula and the left midcingulate cortex that was significantly correlated with an increase in back pain after endotoxin injection and tended to be related to increased sickness. Moreover, recent studies using task-related whole brain fMRI have displayed alterations in the neural processing of social stimuli or rewards, involving subregions of M-FPN and M-CIN, during systemic inflammation [104, 105, 106, 107]. Subregions of the M-FPN have been shown to correlate with circulating levels of interleukin-6 in healthy adults after endotoxin injection [102, 104, 108]. At the same time, the M-FPN consists of cortical and subcortical regions, many of which are associated with parasympathetic regulation [57]. This makes it an ideal candidate for a central nervous region mediating direct neural modulation of immune cells or immune organs as reported by previous studies [3, 4, 25].

Our study has 2 major limitations. First of all, we did not use a sham control for the endotoxemia. Thus, we cannot completely rule out that some of the effects would have equally shown after a saline injection. We did partially control for expectation, though, as our participants thought they would receive endotoxin or saline and, furthermore, did not know at which time point the injection would occur (the intravenous line was in place the whole experiment). In theory, the increase of serum ACTH observed in our study may have been influenced by stress from the injection, blood sampling, or the noisy fMRI environment. However, the shape of the ACTH time course that we have been able to observe with high temporal resolution (shown in Fig. 2f) is quite different from a normal response to acute stress (cf. [109], shown in Fig. 2). In contrast, our ACTH time course is much more similar to previous studies using the human endotoxemia model [110, 111].

Second, our sample size is rather small for an fMRI study. It is therefore best to assume that our results cannot be generalized but only describe the behaviour of the studied sample (much like the earlier fixed-effect analyses in fMRI). However, the sample size is only one of the several factors influencing statistical power [112]. We therefore tried our best to mitigate this limitation by using highly precise data acquisition and preprocessing, collecting as much data per subject as possible (about 80 min of fMRI), and using advanced noise correction methods that have previously been shown to improve the signal-to-noise ratio [46, 47, 50, 51]. Furthermore, we also benefited from a comparison with our previous studies, which used a very similar methodology but with a larger sample size [52, 55]. Finally, we focused on statistical analyses with the highest power (one-sample *t* tests) and avoided detailed interpretation of higher order statistics (e.g., connectivity changes).

In conclusion, we identified 6 hypothalamic regions associated with inflammation in humans and studied their connection with large-scale brain networks. Measuring how the hypothalamus detects or modulates systemic inflammation is a first step to understand central immunomodulation. Furthermore, the results can be translated directly to human patients suffering from inflammatory diseases.

## Statement of Ethics

The study was conducted in accordance with the Declaration of Helsinki. It was approved by the Local Ethics Committee of Hannover Medical School (IORG0002700, approval No. 7427). All participants gave written informed consent.

## Conflict of Interest Statement

The authors have no conflicts of interest to declare.

## Funding Sources

This work was supported by the Horst Görtz Foundation.

## Author Contributions

N.F. assisted with study design, methodology optimization, data acquisition and curation, data preprocessing and analysis, discussion of results, results visualization, and writing of the manuscript. J.M. assisted with data acquisition, data preprocessing and analysis, discussion of results, results visualization, and critical review of the manuscript. M.M. was involved in study design and data acquisition. N.F. assisted with data acquisition. F.B. assisted with study design and supervision, methodology optimization, data acquisition and analysis, discussion of results, results visualization, and writing of the manuscript.

## Data Availability Statement

The raw data as well as necessary scripts for reproducing the results of this study are openly available at Zenodo: N. Färber, J. Manuel, M. May, N. Foadi, F. Beissner. (2021). fMRI study of experimental endotoxemia in humans (Version 1.0) [Data set]. Zenodo. http://doi.org/10.5281/zenodo.5084809.

## Figures and Tables

**Table 1:** Hypothalamic regions associated with inflammation

Region	Correlation with blood serum (*r*)		Cluster size	Max *t* value	Side	MNI coordinates, mm			Hypothalamic nuclei
	TNF	ACTH				*x*	*y*	*z*	
1: Figure 4c	−0.94	−0.93	192	11.9	L	−8	0	−13	Supraoptic nucleus, LH area, and anterior hypothalamic area
			
			41	6.14	L	−4	−4	−7	LH area

2: Figure 4d		−0.90	169	26.8	L	−3	−2	−11	Paraventricular nucleus, dorsomedial hypothalamic nucleus, and anterior hypothalamic area

3: Figure 4E	0.89	0.88	192	29.3	L	−6	−1	−10	LH area and anterior hypothalamic area
			
			19	4.62	L	−1	−4	−6	Paraventricular nucleus
			
			6	3.01	L	−2	−7	−12	Supramammillary nucleus

4: Figure 4F	−0.84	−0.90	181	26.5	R	4	−8	−8	Posterior hypothalamic area, supramammillary nucleus, and LH area
			
			10	3.44	R	6	−4	−16	Tuberomammillary nucleus

5: Figure 4g		−0.89	104	21.1	L	−2	−8	−10	Supramammillary nucleus, mammillary bodies, and posterior hypothalamic area

6: Figure 4h	−0.89	−0.97	68	10.6	R	3	0	−3	Bed nucleus of the stria terminalis and paraventricular nucleus

TNF, tumour necrosis factor; ACTH, adrenocorticotropic hormone; MNI, Montreal Neurological Institute; LH, lateral hypothalamic.

**Table 2:** Large-scale networks involved in functional connectivity with the established hypothalamic regions

Large-scale network involved brain regions	Cluster size	Max *t* value	Side	MNI coordinates, mm		
				*x*	*y*	*z*
*Network of subregion 1 (medial frontoparietal network) (Fig. 4c)*						

Frontal pole	447,168	16.8	L	−10	−3	−17
Lateral occipital cortex						
Temporal pole						
Praecuneus cortex						
Superior frontal gyrus						
Middle temporal gyrus						
Paracingulate gyrus						
Cingulate gyrus						
Cerebellum (left crus I)						
Cerebellum (right crus I)						

Middle frontal gyrus	6,776	6.04	R	43	30	22
Inferior frontal gyrus						
Frontal pole						

*Network of subregion 2 (occipital-pericentral network) (Fig. 4d)*						
Lateral occipital cortex	640,562	10.1	R	13	−5	−13
Postcentral gyrus						
Precentral gyrus						
Precuneus cortex						
Frontal pole						
Occipital pole						
Lingual gyrus						

*Network of subregion 3 (occipital-pericentral network) (Fig. 4e)*						
Postcentral gyrus	367,475	7.39	R	4	5	−11
Precentral gyrus		6.16	R	69	−12	−21
Occipital pole						
Lingual gyrus						
Lateral occipital cortex						
Precuneus cortex						
Superior parietal lobule						
Occipital fusiform gyrus						

Parahippocampal gyrus	4,132	12.2	L	−8	−9	−14

Left caudate	1,438	4.29	L	−24	1	13
Left putamen						

Frontal pole	538	5.89	R	9	71	14

Frontal pole	277	5.07	R	11	62	−2

*Network of subregion 4 (principal regions of the midcingulo-insular network) (Fig. 4f)*						
Precuneus cortex	7,558	5.29	R	5	−43	43
Cingulate gyrus		5.12	L	−8	−59	50
Frontal pole	4,605	4.66	R	33	31	51
Middle frontal gyrus						

Superior frontal gyrus	517	4.33	R	30	6	70

Cingulate gyrus	396	4.69	L	−1	40	12
Paracingulate gyrus						

Cingulate gyrus	243	4.86	R	6	−5	31

*Network of subregion 5 (midcingulo-insular network) (Fig. 4g)*						
Precentral gyrus	234,148	6.18	R	47	2	3
Postcentral gyrus		5.77	L	−61	−3	43
Supramarginal gyrus						
Frontal pole						
Cingulate gyrus						
Central opercular cortex						
Insular cortex						
Juxtapositional lobule cortex						
Middle frontal gyrus						
Superior parietal lobule						

Inferior temporal gyrus	794	5.12	L	−50	−62	−21
Mammillary body						
Lateral occipital cortex						
Temporal occipital fusiform cortex						
Middle temporal gyrus						
Occipital fusiform gyrus						

*Network of subregion 6 (principal regions of the midcingulo-insular network) (Fig. 4h)*						
Precentral gyrus	587,030	16.5	R	6	0	3
Frontal pole						
Praecuneus cortex						
Postcentral gyrus						
Lateral occipital cortex						
Right thalamus						

MNI, Montreal Neurological Institute.
